# An Updated Overview of the Role of CYP450 during Xenobiotic Metabolization in Regulating the Acute Myeloid Leukemia Microenvironment

**DOI:** 10.3390/ijms24076031

**Published:** 2023-03-23

**Authors:** Cristian Sandoval, Yolanda Calle, Karina Godoy, Jorge Farías

**Affiliations:** 1 Escuela de Tecnología Médica, Facultad de Salud, Universidad Santo Tomás, Los Carreras 753, Osorno 5310431, Chile; 2 Departamento de Ingeniería Química, Facultad de Ingeniería y Ciencias, Universidad de La Frontera, Temuco 4811230, Chile; 3 Departamento de Ciencias Preclínicas, Facultad de Medicina, Universidad de La Frontera, Temuco 4811230, Chile; 4 School of Life and Health Sciences, University of Roehampton, London SW15 4JD, UK; yolanda.calle-patino@roehampton.ac.uk; 5 Núcleo Científico y Tecnológico en Biorecursos (BIOREN), Universidad de La Frontera, Temuco 4811230, Chile; karina.godoy@ufrontera.cl

**Keywords:** adults, cytochrome, monooxygenase, polymorphisms

## Abstract

Oxidative stress is associated with several acute and chronic disorders, including hematological malignancies such as acute myeloid leukemia, the most prevalent acute leukemia in adults. Xenobiotics are usually harmless compounds that may be detrimental, such as pharmaceuticals, environmental pollutants, cosmetics, and even food additives. The storage of xenobiotics can serve as a defense mechanism or a means of bioaccumulation, leading to adverse effects. During the absorption, metabolism, and cellular excretion of xenobiotics, three steps may be distinguished: (i) inflow by transporter enzymes, (ii) phases I and II, and (iii) phase III. Phase I enzymes, such as those in the cytochrome P450 superfamily, catalyze the conversion of xenobiotics into more polar compounds, contributing to an elevated acute myeloid leukemia risk. Furthermore, genetic polymorphism influences the variability and susceptibility of related myeloid neoplasms, infant leukemias associated with mixed-lineage leukemia (*MLL*) gene rearrangements, and a subset of de novo acute myeloid leukemia. Recent research has shown a sustained interest in determining the regulators of cytochrome P450, family 2, subfamily E, member 1 (*CYP2E1*) expression and activity as an emerging field that requires further investigation in acute myeloid leukemia evolution. Therefore, this review suggests that *CYP2E1* and its mutations can be a therapeutic or diagnostic target in acute myeloid leukemia.

## 1. Background

Acute myeloid leukemia is a cancerous condition that affects hemopoietic stem cells or progenitors and is defined by the stopping of myeloid lineage development and abnormal proliferation [1]. The most prevalent acute leukemia in adults is acute myeloid leukemia, which has a wide range of genetic variations. In 2021, more than 20,000 new cases of acute myeloid leukemia were expected in the US [2]. Traditionally, acute myeloid leukemia has been categorized based on immunophenotype and morphology. However, genetic abnormalities, such as chromosomal translocations and transcription factor involvement, must be considered in acute myeloid leukemia diagnostic algorithms [3,4]. These factors led to the classification of acute myeloid leukemia into six groups [3]: myeloid proliferations linked to Down syndrome, myeloid sarcoma, recurring genetic abnormalities, therapy-related myeloid neoplasms, and acute myeloid leukemia with myelodysplasia-related alterations.

Oxidative stress is implicated in several acute and chronic diseases, including hematological malignancies such as acute myeloid leukemia, which is the most common acute leukemia in adults, with an increasing incidence with age and high relapse rates [1]. Despite current advancements in the treatment of acute myeloid leukemia, refractory disease remains prevalent, with disease relapse being the major cause of treatment failure [5]. The current acute myeloid leukemia management guidelines largely rely on high-dose chemotherapy with cytarabine- and anthracycline-based regimes and allogeneic hematopoietic stem cell transplantation [6].

Cytarabine with anthracycline induction therapy is the standard of care for acute myeloid leukemia. The most commonly used regimen includes anthracycline daunorubicin (45–90 mg/m^2^) on days 1–3 and cytarabine (100–200 mg/m^2^) in continuous infusion on days 1–7 [7]. In this regard, systematic reviews have assessed the efficiency of cytarabine and daunorubicin regimens and determined that 62.1% of patients achieve full remission. In addition, it has been noted that, when cytarabine or daunorubicin doses are raised throughout treatment, the rate of full remission increases [8]. However, the dose intensification of cytarabine, such as doses of 1–2 g/m^2^/12 h [8,9] or the extension of the treatment duration to 10 days [10], did not result in improved results and was associated with increased toxicity [8,9].

Even in individuals who receive doses of myeloablative chemotherapy or radiation given for hematopoietic stem cell transplantation, the malignant stem cell might undergo further mutations [11]. The graft-versus-leukemia effect, which results from the elimination of these stem cells by T- and natural killer (NK) lymphocytes of the donor, is one of the advantages of allogeneic hematopoietic stem cell transplantation [12,13]. However, the presence of comorbidities greatly compromises the results of hematopoietic stem cell transplantation, as a result of which this therapeutic option is not recommended for these patients [14].

**Figure 1:**
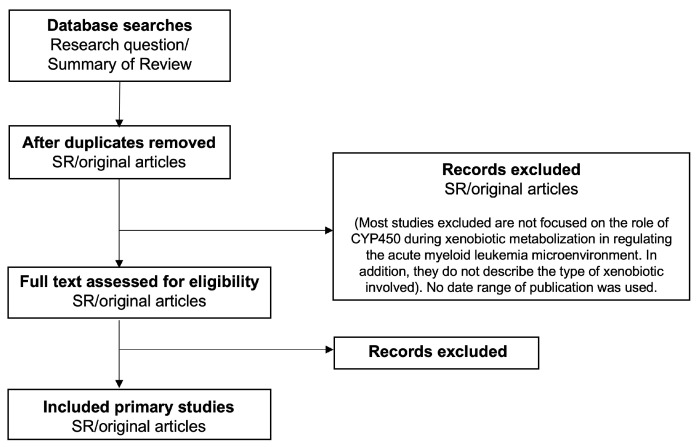
Flow diagram.

Acute myeloid leukemia treatment is becoming more customized based on the molecular characteristics of the disease because of better knowledge of its pathophysiology [5,15]. This allows for greater risk assessment and more personalized drugs. Seven of the nine novel drugs approved for the treatment of relapsed or refractory acute myeloid leukemia (R/R AML)—azacitidine, enasidenib, glasdegib, gemtuzumab ozogamicin, gilteritinib, low-dose cytarabine, midostaurin, venetoclax and, venetoclax plus low-dose cytarabine—act via a molecularly defined target, as opposed to standard cytotoxic chemotherapy [16,17,18,19,20,21,22]. There are still certain unmet needs, despite the development and popularity of these new medicines for the treatment of acute myeloid leukemia. These include the fact that many individuals with R/R AML who do not currently have targetable mutations still have few therapy choices. Aside from the limited percentage of patients who continue with an allogeneic hematopoietic cell transplant, none of the recently approved medicines are curative [23]. Therefore, this article reviews current acute myeloid leukemia pathogenesis and novel therapies. Figure 1 illustrates the flow chart for the study selection process.

## 2. Pathophysiology of Acute Myeloid Leukemia

### 2.1. Cytogenetic Abnormalities

Acute myeloid leukemia is characterized by mutations in hematopoiesis-related genes [24]. Ineffective erythropoiesis and bone marrow failure are caused by these mutations, which cause a clonal increase in undifferentiated myeloid progenitors (blasts) in the peripheral blood and bone marrow. Recent research has suggested that it could result from several recurring genetic changes in hematopoietic stem cells that accumulate over time [15,25,26,27,28,29,30]. Acute myeloid leukemia often develops from scratch in a previously healthy person. Although the precise source of genetic abnormalities is unknown, a few known risk factors include smoking, chemotherapy, and radiation exposure [31]. Aplastic anemia, paroxysmal nocturnal hemoglobinuria, myelodysplastic syndrome, and myeloproliferative diseases can all develop into acute myeloid leukemia [32,33].

Genetic mutations that have familial causes should also be considered (Table 1). The most prevalent mutational subset in acute myeloid leukemia is type 1 mutations, which are present in about two-thirds of patients and result in abnormal activation and proliferation of cellular signaling pathways (e.g., FMS-like tyrosine kinase 3 (*FLT3*); Ki-ras2 Kirsten rat sarcoma viral oncogene homolog (*KRAS*); NRAS Proto-Oncogene, GTPase (*NRAS*); Tyrosine-protein phosphatase non-receptor type 11 (*PTPN11*); neurofibromin 1 (*NF1*); and KIT proto-oncogene, receptor tyrosine kinase (*KIT*)). It is interesting to note that mutations in this class are usually found in subclonal cellular fractions, indicating that they are frequently late clonal events in the development of illness [15].

Somatic mutations within key epigenetic regulators are identified in >50% of acute myeloid leukemia, and are now recognized as a key, and often an inciting, component of leukemogenesis [15]. It is of interest that age-related clonal hematopoiesis, identified in >10% of individuals over age 65, is predominantly defined by the clonal outgrowth of preleukemic clones harboring mutations in one of the genes within this epigenetic class [34,35].

Along with *FLT3* and DNA methyltransferase 3 alpha (*DNMT3A*; https://www.ncbi.nlm.nih.gov/gene/1788; accessed on 14 January 2023), nucleophosmin 1 (*NPM1*) is one of the three most frequent driver mutations in acute myeloid leukemia. The regulation of pathways for cell proliferation, differentiation, adhesion, and death by receptor tyrosine kinase (RTKs) signaling pathways in acute myeloid leukemia is crucial for the onset and spread of malignancy. Around 20 separate subfamilies make up the RTKs, which include class III and *TYRO3*, *AXL*, and *MERTK* (TAM) family RTKs [53]. Class III RTKs, which include *c-Kit*, *CSF1R*, *FLT3*, and platelet-derived growth factor receptors (PDGFR), have been discovered to have a major effect on leukemogenesis and transformation into acute myeloid leukemia. Class III RTKs have been linked to aberrant activation that promotes proliferation in leukemia. Particularly, *FLT3* expression and c-Kit mutations are critical for acute myeloid leukemia. Both RTKs have been crucial targets in the development of antileukemic therapies, since they are both associated with worse prognoses. *TYRO3*, *AXL*, and *MERTK* are members of the TAM family of RTKs. These are essential for platelet activation and stabilization, for the normal hematopoiesis of certain innate immune cells, and have been linked to erythropoiesis [37]. TAM RTKs are critical for normal hematopoietic development, but they can activate pathways for proliferation and survival in cancer cells, particularly in acute leukemia [54]. TAM RTKs, particularly *AXL* and *MERTK*, have been linked to hematologic malignancies and have grown in interest as potential targets for creating novel treatments [54,55,56,57,58].

In addition, compared to the genomes of other cancers, acute myeloid leukemia genomes are typically less mutated [59], with similar distributions of mutations before and following relapse [60,61]. Of these mutations, many frequently occur in genes involved in DNA methylation and epigenetic regulation, such as *DNMT3A*, *TET1/2*, and *IDH1/2* [62]. Hassan et al. [63] point to a greater need for understanding acute myeloid leukemia through a non-genetic lens, focusing on DNA methylation and other epigenetic modalities. They also suggest relative independence between the progression of acute myeloid leukemia and the disease’s strictly genetic landscape.

*IDH1* gene mutations are present in around 6–10% of individuals with acute myeloid leukemia [64]. Given its capacity to create cytoplasmic NADPH and glucose sensing, *IDH1* is implicated in controlling cellular metabolism, particularly lipid metabolism [65,66]. Isocitrate is oxidized to α-ketoglutarate by wild-type isocitrate dehydrogenases [67]. It has been hypothesized that the *IDH1* Arg132 mutation alters the way the enzyme functions, causing α-ketoglutarate to be converted to R(—)-2-hydroxyglutarate [68]. This excess of R(—)-2-hydroxyglutarate causes cellular proliferation to rise and cellular differentiation to be compromised [69].

Nucleophosmin 1 mutations, occurring almost exclusively within exon 12 of the gene, occur in approximately one-third of adults with acute myeloid leukemia, and in more than 50% of NK-AML. The *NPM1* gene encodes for the nuclear chaperone protein NPM, which shuttles between the nucleus and cytoplasm and plays a role in diverse cellular functions, including protein formation, ribosome biogenesis, DNA replication, and the cell cycle. *NPM1* mutations are typically stable throughout the disease course, are identified in nearly all leukemic cells, and impart a distinct expression profile [70]. *NPM1* mutations in the setting of mutant *DNMT3A*, particularly in the setting of *FLT3* internal tandem duplication (FLT3-ITD), confer a markedly poor prognosis [15,71].

Approximately 20% to 25% of adults with acute myeloid leukemia have mutations involving the myeloid transcription factors Runt-related transcription factor 1 (*RUNX1*), *CEBPA*, and/or GATA binding protein 2 (*GATA2*) [24]. In this sense, *RUNX1* is known to act as a direct transcriptional activator of several proteins important for platelet function and as a transcriptional repressor of others, including *MYH10* [72,73,74,75] and *ANKRD26* [76]. In addition, *CEBPA* encodes a master hematopoietic transcription factor that acts as a critical regulator of granulocyte and monocyte differentiation [77], while *GATA2* encodes a zinc finger transcription factor critical for normal hematopoiesis [78,79] and lymphatic vascular development [80,81].

Tumor protein p53 (*TP53*) is a key tumor suppressor with highly variable functions related to the maintenance of genomic stability, including regulation of cellular senescence, apoptosis, metabolism, and DNA repair. Although uncommon in de novo acute myeloid leukemia, *TP53* mutations occur in ~15% of therapy-related acute myeloid leukemia or acute myeloid leukemia with myelodysplastic syndrome-related changes, and are predominantly associated with complex cytogenetics, advanced age, chemotherapy resistance, and poor survival [47,48]. Irrespective of age or treatment modality, *TP53* mutations in acute myeloid leukemia portend lower response rates and inferior outcomes compared with *TP53* wild-type acute myeloid leukemia patients [49].

Frequently mutated in myelodysplastic syndrome and myeloproliferative neoplasms, mutations in splicing factors (*SF3B1*, *SRSF2*, *U2AF1*, and *ZRSR2*) are identified in ~10% of patients with acute myeloid leukemia and are associated with older age, less proliferative disease, poor rates of response to standard treatment, and decreased survival. Spliceosome mutations are postulated to promote malignancy through the missplicing of various genes involved in epigenetic regulation, transcription, and genome integrity [50].

The structural maintenance of chromosomes (*SMC3* and *SMC1A*), RAD cohesin complex component (*RAD21*), and cohesin subunit SA (*STAG1/STAG2*) make up the four core elements of cohesin with a ring shape. Cohesin helps several other subunits, such as *NIPBL*, *MAU2*, *WAPL*, *PDS5A*, *PDS5B*, and sororin, to form cohesion during the cell cycle [82,83,84,85]. Consequently, the ring-shaped cohesin controls the sister chromatids’ separation, DNA replication, and repair of the broken double-strand DNA during the advancement of the cell cycle [86,87,88,89,90]. To control chromatin structure and gene expression, the cohesin complex can also interact with the transcriptional repressor CTCF, promoters, mediators, enhancers, initiation and elongation forms of RNA polymerase II (*RNAPII*), or transcription factors (TFs) [87,91,92,93,94,95].

Acute myeloid leukemia prognosis is exceedingly variable and unpredictable. It can be caused by molecular changes, chromosomal translocations, or genetic mutations. Genetic mutations have been shown to occur in around 97% of cases. Table 2 shows an updated classification of acute myeloid leukemia based on the National Comprehensive Cancer Network’s cytogenetic and molecular criteria [96,97]. The following are examples of cytogenetic subsets: (1) chromosomal translocations [t(15;17)(q22,q21)] and core binding factor acute myeloid leukemia (CBF-AML), both of which are cytogenetic/molecular subgroups of inversion 16 [inv16(p13;q22)] or t(16;16)(p13;q22); (2) individuals with cytogenetically normal acute myeloid leukemia (CN-AML) who have monosomy 5 or 7 or t(9 or 11) have a low risk (40–50% of patients) [85,86,87,88,89,90]; (3) individuals with t(6;9), inv (3), or 11q changes (11q23 translocations) [85,86,87,88,89,90]; (4) and those with other karyotypes have been demonstrated to have a higher risk of treatment failure and mortality [98,99,100,101,102,103]. Furthermore, people with translocations involving the *MECOM* (myelodysplastic syndrome-1 and ecotropic viral integration site 1 (EVI1) complex locus) gene on chromosome 3q26.2 have a very poor prognosis [104,105].

### 2.2. Mutations

**Figure 2:**
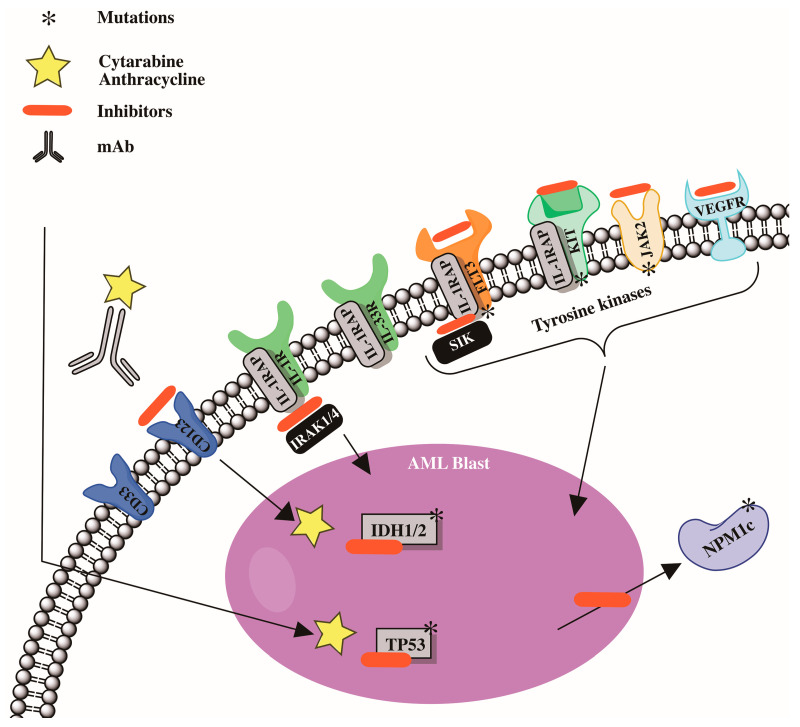
Cytogenetic abnormalities and mutations involved in development in acute myeloid leukemia (AML).

Modern molecular technologies have permitted the identification of a wide spectrum of genetic disorders. Six genes have already been incorporated into the European Leukemia Net risk categories [5], including *FLT3*, *NPM1*, CCAAT/enhancer binding protein α (*CEBPA*), *RUNX1*, additional sexcombs-like 1 (*ASXL1*), and *TP53* [5]. Other recurrent gene mutations in acute myeloid leukemia patients have been discovered [106,107,108,109,110,111]. Furthermore, research has been undertaken on the essential roles played by recurrent gene mutations in the pathogenesis of acute myeloid leukemia, as well as the development of medicines that precisely target gene alterations [112,113,114,115,116,117,118]. Preleukemic cell detection, particularly in acute myeloid leukemia patients with mutant *DNMT3A* and *TET2* genes, has been linked to leukemia genesis [119,120]. Mutations in the *DNMT3A* and *TET2* genes are common in patients with clonal hematopoiesis of undetermined potential [34,35,121,122]; these mutations may serve as markers for the identification of preleukemia cells [119,120]. A summary of the pathophysiology of acute myeloid leukemia is shown in Figure 2.

## 3. Acute Myeloid Leukemia Microenvironment

Currently, niches are thought of as microenvironments that mix non-hematopoietic cells with the structure of the bone marrow to encourage hematopoietic stem cell self-renewal and differentiation by offering beneficial and crucial components [123,124]. The non-hematopoietic progenitors known as mesenchymal stromal cells are an essential component of the bone marrow niche.

Due to their ability to directly govern the development and differentiation of hematopoietic stem cells and their ability to release a range of soluble growth factors and cytokines, mesenchymal stromal cells really play an important role in immunomodulation [125,126,127]. Mesenchymal stromal cells express significant hematopoietic factors, such as stem cell factor and stromal cell-derived factor 1. Additionally, they give off trophic factors that control the immune system and turn on the body’s own stem cells to repair damaged tissues [128,129,130,131].

The ability of allogeneic stem cells to differentiate into various stromal marrow components, such as pericytes, bone marrow stromal cells, myofibroblasts, osteoblasts, osteocytes, and endothelial cells, is also essential for allogeneic stem cell transplantation to be successful [132,133]. Bone marrow mesenchymal stromal cells are commonly recognized as significant contributors to tumor genesis, recurrence, and treatment resistance in the context of acute myeloid leukemia due to their ability to provide leukemic blasts with survival and anti-apoptotic signals [134,135]. According to several studies, co-culturing acute myeloid leukemia blasts with stromal or mesenchymal stem cells has been linked to increased in vitro tumor cell viability [136,137], aberrant phenotypic expression [138,139,140], and decreased chemoresistance [134,141,142].

Additionally, animal models of myeloproliferative neoplasms [143,144,145,146,147], myelodysplastic syndrome [148,149], and acute myeloid leukemia [150,151] have been used to illustrate niche-induced disease onset in vivo. Ex vivo expanded mesenchymal stromal niche cells from myelodysplastic syndrome, and acute myeloid leukemia patients, have been shown to have a variety of functional and molecular alterations, including chromosomal aberrations [152,153], transcriptional changes [154], and epigenetic changes [155], as well as functional changes in their ability to differentiate and hematopoietic stem cell-supportive behaviors [156,157].

By using array comparative hybridization and transcriptome profiling, it was also discovered that CD271+ mesenchymal stromal cells directly obtained from myelodysplastic syndrome patients had genetic and transcriptomic changes [158,159,160]. The down-regulation of dicer 1, ribonuclease III (DICER), and SBDS ribosome maturation factor (SBDS) [149,157,160] and the activation of beta-catenin in osteoblastic cells are two processes that have been proven to start malignant transformation in mice and have also been documented in patient-derived mesenchymal cells [150]. More recently, it was discovered that mice with nestin+ cells developed juvenile myelomonocytic leukemia when a *PTPN11* mutant was expressed [147].

Despite the above-mentioned, few studies have used environmental sampling [161,162] and biomarkers such as malondialdehyde, total antioxidant capacity, thiobarbituric acid reactive substances, protein carbonyl, and lipid hydroperoxide evaluation [163,164,165,166] to better characterize chemical exposures, which could provide powerful insights to better understand the continuum between routes of exposure, chemical body burden, and risk of acute myeloid leukemia. Several studies have found that gene polymorphisms in xenobiotic pathways, such as cytochrome P450 family 2 subfamily E member 1 (*CYP2E1*), glutathione S-transferase Mu 1 (*GSTM1*), NAD(P)H: quinone oxidoreductase (*NQO1*), N-acetyltransferase 2 (*NAT2*), and multidrug resistance protein 1 (*MDR1*), influence leukemia risk alone or in combination with chemical exposure [166].

## 4. Mechanisms of Liver and Bone Marrow *CYP2E1* Induction, Activity, and Degradation

CYPs are a family of heme-containing proteins that play an essential function in the metabolism of a wide variety of xenobiotics [167]. CYP450 proteins have an essential function in tumorigenesis by activating or deactivating carcinogens, which influences tumour start and progression [168]. Recent research has demonstrated that *CYP2E1* is not only markedly elevated in the liver, but also expressed in bone marrow [169]. Drugs and plants (isoniazid, *Salvia miltiorrhiza*, *Schisandra chinensis*), pollutants (phenylamine), food additives (coffee and cocoa polyphenols), and industrial material and environmental contaminants (benzene) can stimulate *CYP2E1* activity [170,171].

Benzene has been recognized as an environmental contaminant that can generate hematotoxicity and leukemogenicity [172,173,174]. Many studies have hypothesized that the conversion of benzene to reactive metabolites by hepatic enzymes, specifically *CYP2E1*, is a precondition for the cyto- and genotoxic effects of benzene exposure [175,176,177]. Hydroquinone, phenol, trans–trans muconic acid, and catechol are the principal benzene metabolites [178]. These phenolic metabolites work synergistically to increase benzene toxicity [179,180,181]. In terms of the mechanism of its toxicity and carcinogenicity, this process of multimetabolite genotoxicity is another distinguishing feature of benzene compared to other compounds. Thereafter, benzene metabolites undergo further activation by myeloperoxidase, which is abundant in bone marrow tissue. Inducing not only hemopoietic cellular damage [182,183,184] but also bone marrow stromal cell dysfunction [185].

Among the biochemical mechanisms abnormally elevated in malignancies, including acute myeloid leukemia, is the phosphoinositide 3-kinase-Akt-mammalian target of rapamycin route (PI3K-Akt-mTOR pathway) [186]. PI3K enzymes have crucial functions in cell metabolism, proliferation, and survival [187,188]. PI3K activation triggers pathways of signaling that stimulate cell differentiation, metabolism, migration, proliferation, and survival [189]. Ethanol-induced suppression of Akt phosphorylation and pharmacological modulation of Akt can result in *CYP2E1*-induced hepatic oxidative stress, which could be a viable treatment for ethanol-induced fatty liver [190,191]. Therefore, the effect of alcohol on *CYP2E1* induction and the involvement of PI3K/Akt in guarding against the cytotoxicity of *CYP2E1* suggest that *CYP2E1* overexpression may reduce the expression of critical proteins in the *PI3K* signaling pathway [190,191].

## 5. Mechanisms of CYP-Mediated Carcinogenesis and the Roles of Their Isoforms

CYP450 enzymes serve a critical role in preventing oxidative damage; they metabolize several exogenous and endogenous genotoxic chemicals, such as hydrogen peroxide, by inserting an oxygen atom into the substrate [192].

Specific single-nucleotide polymorphisms at the *CYP450* loci (*CYP2D6*, *CYP1A1*, *CYP3A5*, and *CYP2E1*) may very well be categorized as risk factors for numerous kinds of cancers, due to the inactivation of enzymatic activity [193,194,195,196,197,198,199,200].

At the *CYP2B6* gene locus, the G516T polymorphism has been recognized as a nonsense polymorphism that decreases the activity of the protein complex. Hence, people who have the T allele (TT) have a reduced enzymatic activity than people who have the wild-type G allele (GG), but individuals holding the genotype GT exhibit intermediate activity [201].

**Figure 3:**
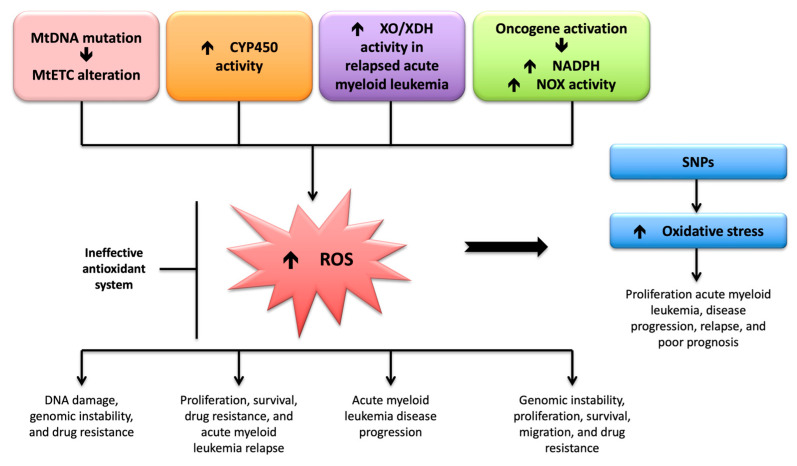
Processes involved in reactive oxygen species production during acute myeloid leukemia cells and their significance in leukemogenesis. Abbreviations: mDNA, mitochondrial DNA; mETC, mitochondrial electron transport chain; NADPH: Nicotinamide adenine dinucleotide phosphate; NOX: NADPH oxidase; XO, xanthine oxidase; XDH, xanthine dehydrogenase.

Ethnicity causes *CYP3A* enzyme activity to vary up to 50-fold. Most genetic variants in the *CYP3A4* gene result in lower enzyme activity. Single-nucleotide polymorphisms in the *CYP3A4* promoter region (A290G) (i.e., CYP3A4*1B) have been implicated as a possible cause of this variability; hence, it may be considered a risk factor for cancer. However, the implications of these single-nucleotide polymorphisms are not well-understood [202,203,204,205]. Hence, diverse allelic expressions of the *CYP450* gene have distinct pathogenic effects and prognostic characteristics in various hematological cancers. Figure 3 shows the main processes involved in ROS production during acute myeloid leukemia.

## 6. Xenobiotics and CYP450 Activation

The broad category of xenobiotics includes substances that are generally safe but may be harmful, such as medications, environmental toxins, cosmetics, and even elements included in our diet, such as food additives [206,207,208,209]. Storage of xenobiotics can serve as a defense mechanism or a way for bioaccumulation to result in harmful consequences. The physiologic connection between the storage depot and the target tissues for a particular toxin determines this possible hazardous pathway [206,208].

Xenobiotic metabolism increases their water solubility, thus enhancing their elimination from the body [210]. When xenobiotics are consumed orally, they go through the upper gastrointestinal tract and, if they are absorbed, are then transferred to the liver via the hepatic portal vein. The liver chemically converts both endogenous and exogenous substances, utilizing the CYP450 family of enzymes [211,212]. In fact, in the absorption, metabolism, and cellular excretion of xenobiotics, three steps may be distinguished: (i) inflow by transporter enzymes, (ii) phases I and II, mediated by drug-metabolizing enzymes, and (iii) phase III, the excretion mediated mainly by transporter enzymes [195,206,213]. Non-metabolized and unexcreted xenobiotics build up in the body and can cause chronic illnesses and inflammation [214].

The inflow of xenobiotics is mediated by sodium taurocholate cotransporting polypeptide, organic anion transporting polypeptides, and organic anion transporters [195]. Phase I enzymes, such as those in Cytochrome P450 (CYP450), flavin-containing monooxygenases (FMOs), monoamine oxidases (MAOs), and xanthine oxidase/aldehyde oxidase (XO/AO) superfamily, catalyze the conversion of predominantly lipophilic xenobiotics into more polar compounds by oxidation, reduction, or hydrolysis [115,116,117,167]. Phase I processes that introduce polar groups create the sites needed for conjugation reactions, which are carried out by Phase II enzymes [118,119,120,121,122,123,124,125,126,127,128,129,130,131,132,133,134,135,136,137,138,139,140,141,142,143,144,145,146,147,148,149,150,151,152,153,154,155,156,157,158,159,160,161,162,163,164,165,166,167,168,169,170,171,172,173,174,175,176,177,178,179,180,181,182,183,184,185,186,187,188,189,190,191,192,193,194,195,196,197,198,199,200,201,202,203,204,205,206,207,208,209,210,211,212,213,214,215,216,217,218,219,220]. Phase I metabolites are frequently conjugated with glucuronic acid, glutathione, sulfate, amino acids, or methyl or acetyl groups, even though Phase II enzymes can directly operate on the parent substance [221]. The addition of these large anionic groups, which may detoxify reactive electrophiles (either parent chemicals or Phase I metabolites), results in Phase II metabolites with enhanced hydrophilicity and molecular weight, which cannot penetrate the phospholipid membrane barrier [115,172,221,222,223]. The anionic groups of phase III xenobiotic transporters operate as affinity tags for a variety of membrane carriers belonging to two major clusters: ATP binding cassette transporters, including the multidrug resistance protein family, and solute carrier transporters [224,225,226].

There are significant inter- and intra-individual differences in the ability to metabolize, detoxify, and expel xenobiotics. These are genetic, epigenetic, environmental, and physiological pathophysiological in nature, and they change during life [215,225,227,228,229,230]. Most xenobiotics are detoxified and eliminated via a complicated network of numerous enzymes and pathways. The interaction of xenobiotic local or cellular concentration, specific enzyme affinity, tissue-specific enzyme expression, stability, and cofactor availability frequently determines which metabolic processes prevail at any given time [167,206,231].

## 7. *CYP2E1* Expression and Regulation in Acute Myeloid Leukemia

*CYP2E1* is a hepatic monooxygenase involved in the metabolism of xenobiotics. The *CYP2E1* gene is linked to the metabolism of many carcinogens. *CYP2E1* is essential for the metabolism of endogenous compounds (such as acetone and fatty acids) as well as external substrates such as medications, contaminants, and ethanol [232]. The mechanism of both *CYP2E1*-mediated metabolism (e.g., styrene metabolism) and enzyme-associated toxicity, such as methemoglobinemia and acetaminophen-induced liver necrosis, has recently piqued researchers’ attention. Electrons are transferred to the substrates through *CYP2E1*-mediated oxidation of reduced nicotinamide, adenine dinucleotide phosphate, and molecular oxygen. This reaction adds additional polar groups to the substrates and generates hazardous intermediates such as epoxides or aldehydes [233].

An in silico approach for simulating the *CYP2E1* active site as a sheet of hexagonal blocks has been devised, which might directly relate to the two-dimensional structure of chemicals. For a core part of the *CYP2E1* active site, a region with the shape of benzopyrene was suggested as a model [234]. This was carried out to predict how the drug would be broken down at both sites and in what order.

*CYP2E1* polymorphisms have been linked to potential mechanisms of tumor initiation. The CYP2E1*5 allele is linked to an increased risk of developing acute myeloid leukemia and acute lymphoblastic leukemia [235]. When lymphocytes with the *CYP2E1* single-nucleotide polymorphisms rs2070673TT and rs2030920CC are exposed to phenol, they exhibit increased transcription and enzyme activity as well as increased DNA damage [236]. The CYP2E1*5B(C-1019T) polymorphism has not been linked to therapy-related acute myeloid leukemia or myelodysplastic syndrome [237]. In individuals with chronic lymphocytic leukemia/small lymphocytic lymphoma, the CYP2E1*07 (rs2070673) allele has been linked to a higher survival rate [238]. As a result, distinct allelic expressions of the *CYP2E1* gene have diverse pathogenic and prognostic consequences in various hematological malignancies. Furthermore, the existence of polymorphisms does not always correspond with the phenotypic functional activity of *CYP2E1*, and total functional evaluation is more accurate than testing for polymorphisms. Increased *CYP2E1* expression has been linked to liver illnesses such as alcoholic hepatitis and non-alcoholic steatohepatitis and is considered to play a role in their etiology [239,240].

Therapy-related myeloid neoplasms [241], infant leukemia associated with mixed-lineage leukemia (*MLL*) gene rearrangements [242], and a subtype of de novo acute myeloid leukemia [243], have low *NQO1* activity. These findings support the idea that common ambient pollutants detoxified by *NQO1* are risk factors for acute leukemia [243]. In fact, the *NQO1* polymorphism had the strongest connection with acute myeloid leukemia and inv(16)/CBF-MYH11 in a study concentrating on de novo acute myeloid leukemia [243]. While the translocation has been postulated to disrupt the *NQO1* gene, which is located on chromosome 16q22.1 [243], it is also possible that myeloid cells with this chromosomal abnormality are more sensitive to environmental pollutants. Furthermore, *CYP2E1* was one of the four most differentially expressed genes in acute myeloid leukemia with inv(16)/CBF-MYH11, being raised 3.3-fold in acute myeloid leukemia with inv(16) [244].

## 8. Common Treatments of Acute Myeloid Leukemia

Between the treatments used for acute myeloid leukemia, we can find azacitidine, which is a pyrimidine nucleoside analog of cytidine that can be directly integrated into RNA, disrupting RNA, protein production, and metabolism [245]. It is only minimally integrated into DNA, covalently linking to DNA methyltransferases and directing their destruction. Without methyltransferases, daughter cells are hypomethylated, and repressed gene expression is reactivated during DNA synthesis [245,246]. Azacitidine, a DNA methyltransferase inhibitor, has been described as restoring tumor suppressor gene function and cell differentiation in patients with myelodysplastic syndrome and acute myeloid leukemia [247].

Azacitidine is available for intravenous and subcutaneous injection, with equivalent absorption for both routes [248]. The cytochrome P450 enzyme, uridine diphosphate, and glutathione transferase do not metabolise azacitidine, according to in vitro investigations. Instead, they are deaminated by cytidine deaminase and excreted primarily through the kidneys [249].

Other treatments for acute myeloid leukemia are based in enasidenib, a novel, mutant IDH2 protein-targeting inhibitor used to treat relapsed or resistant acute myeloid leukemia [250,251,252,253]. Enasidenib reduces the oncometabolite 2-hydroxyglutarate by 90.6% [250,251,252,253]. In vitro research shows that enasidenib inhibits several CYP enzymes and transporters and induces *CYP3A4* [254]. Since enasidenib may induce or inhibit drug-metabolizing enzymes and transporters, the co-administration of enasidenib may increase or reduce the concentrations of combination drugs [254]. However, among patients with relapsed or refractory acute myeloid leukemia, the overall response rate is approximately 40.3% [17].

Finally, drugs against acute myeloid leukemia, such as glasdegib, gilteritinib, midostaurin, cytarabine, and venetoclax, have high oral bioavailability and are widely used [255,256,257,258,259,260]. Most, such as glasdegib, gilteritinib, midostaurin, cytarabine, and venetoclax, are eliminated by oxidative metabolism, mainly *CYP3A4*, with a minor contribution from glucuronidation by uridine diphosphate glucuronosyltransferase 1A [258,259,260,261]. However, because glasdegib, gilteritinib, midostaurin, cytarabine, and venetoclax are a substrate of *CYP3A4* enzyme-mediated metabolism, plasma levels of glasdegib tend to decline; *CYP3A* inhibitors such as ketoconazole should be administered to increase glasdegib levels [256,257,258,259,260,262]. Table 3 shows one-year survival for each drug approved for the treatment of acute myeloid leukemia.

## 9. Carotenoids, CYP2E1 Expression, and Regulation in Acute Myeloid Leukemia

Dietary phytochemicals are one specific class of dietary components having anti-cancer action, among other dietary variables that are well known for their chemo-preventive effects. In addition to being non-essential nutrients, phytochemicals can significantly contribute to the prevention of disease. Numerous phytochemicals have strong anti-oxidant and anti-carcinogenic properties, including polyphenols, flavonoids, allyl sulphides, and carotenoids [271].

Carotenoids are pigments found in fruits, vegetables, and whole grains that are yellow, orange, or red. Patients with asthma, cataracts, and heart disease have been shown to benefit from beta-carotene, the main source of vitamin A and its derivative retinoic acid [272,273,274]. It has also been connected to a lower risk of prostate cancer. While some research has suggested that antioxidants, such as β-carotene, may increase *CYP2E1* activity after moderate alcohol consumption and β-carotene supplementation [275], other studies have found that it is possible to prevent the degree of hepatic steatosis produced by various alcohol doses in order to prevent the progression to more serious injuries [276,277]. Therefore, it is unknown whether β-carotene, when combined with other vitamins, medications, or dietary components, has the capacity to reprogramme epigenetic activity.

## 10. Limitations

The purpose of this review was to describe the pathophysiology of acute myeloid leukemia as well as the role of *CYP2E1* in the xenobiotic metabolism that governs the myeloid leukemia microenvironment. Still, this review found some problems that could make it hard to combine scientific evidence. These problems include: (1) a lack of information because there were so few articles; (2) a lack of *NQO1* levels during *CYP2E1* activity in acute myeloid leukemia; (3) a lack of information about how gene polymorphisms affect the encoded protein; and (4) a lack of thought about how genes and the environment interact.

## 11. Conclusions

Through the conversion of a range of xenobiotics into hazardous intermediates such as reactive oxygen species and free radicals, *CYP2E1* contributes to an elevated acute myeloid leukemia risk. *CYP2E1*-related disorders relate to protein levels, and there are inter-individual variances in *CYP2E1* expression levels, according to research. Furthermore, genetic polymorphism, drugs, plants, pollutants, food additives, and industrial material and environmental contaminants influence the variability and susceptibility to related myeloid neoplasms, infant leukemias associated with *MLL* gene rearrangements, and a subset of de novo acute myeloid leukemia. Recent research has shown a sustained interest in determining the regulators of *CYP2E1* expression and activity as an emerging field that requires further investigation in acute myeloid leukemia evolution. This research has the potential to give insight into novel strategies for the treatment of acute myeloid leukemia via *CYP2E1*.

## Figures and Tables

**Table 1:** Recurrent mutations in acute myeloid leukemia.

Functional Class	Specific Example Mutations	References
Signaling and kinase pathways	FLT3, KRAS, NRAS, KIT, PTPN11, and NF1	[15]
DNA methylation and chromatin modification	DNMT3A, IDH1, IDH2, TET2, ASXL1, EZH2, and MLL/KMT2A	[15,34,35]
Nucleophosmin	NPM1	[36,37]
Transcription factors	CEBPA, RUNX1, and GATA2	[38,39,40,41,42,43,44,45]
Tumor suppressors	TP53	[46,47,48]
Spliceosome complex	SRSF2, U2AF1, SF3B1, and ZRSR2	[49,50]
Cohesin complex	RAD21, STAG1, STAG2, SMC1A, and SMC3	[51,52]

**Table 2:** Classification of acute myeloid leukemia based on the National Comprehensive Cancer Network’s cytogenetic and molecular criteria.

Type	Diagnostic Criteria
Acute myeloid leukemia with minimal differentiation	Blasts are negative (<3%) for MPO and SBB.
	Expression of two or more myeloid-associated antigens, such as CD13, CD33, and CD117.
Acute myeloid leukemia without maturation	≥3% blasts positive for MPO or SBB and negative for NSE.
	Maturing cells of the granulocytic lineage constitute <10% of the nucleated bone marrow cells.
	Expression of two or more myeloid-associated antigens, such as MPO, CD13, CD33, and CD117.
Acute myeloid leukemia with maturation	≥3% blasts positive for MPO or SBB.
	Maturing cells of the granulocytic lineage constitute ≥10% of the nucleated bone marrow cells.
	Monocyte lineage cells constitute <20% of bone marrow cells.
	Expression of two or more myeloid–associated antigens, such as MPO, CD13, CD33, and CD117.
Acute basophilic leukemia	Blasts and immature/mature basophils with metachromasia on toluidine blue staining.
	Blasts are negative for cytochemical MPO, SBB, and NSE.
	No expression of strong CD117 equivalent (to exclude mast cell leukemia).
Acute myelomonocytic leukemia	≥20% monocytes and their precursors.
	≥20% maturing granulocytic cells.
	≥3% of blasts positive for MPO.
Acute monocytic leukemia	≥80% monocytes and/or their precursors (monoblasts and/or promonocytes).
	<20% maturing granulocytic cells.
	Blasts and promonocytes expressing at least two monocytic markers including CD11c, CD14, CD36 and CD64, or NSE.
Acute erythroid leukemia	≥30% immature erythroid cells (proerythroblasts)
	Bone marrow with erythroid predominance, usually ≥80% of cellularity
Acute megakaryoblastic leukemia	Blasts express at least one or more of the platelet glycoproteins: CD41 (glycoprotein llb), CD61 (glycoprotein IIIa), or CD42b (glycoprotein lb)

MPO: myeloperoxidase; NSE: nonspecific esterase–butyrate; SBB: Sudan Black B.

**Table 3:** Drugs approved for the treatment of relapsed or refractory acute myeloid leukemia and the prognoses after treatment.

Drug	One-Year Survival (%)	References
Azacitidine	Complete remission for patients >60 years old: 61.0%	[263]
Azacitidine	45.8%	[264]
Azacitidine	Complete remission, complete remission with incomplete recovery, partial remission in patients >60 years old: 36.2%	[265]
Enasidenib	Complete remission: 19.3%	[261]
Enasidenib	Complete remission with incomplete hematologic recovery: 6.8%	[261]
Enasidenib	Partial remission: 6.3%	[261]
Enasidenib	Morphologic leukemia free state: 8.0%	[261]
Glasdegib	Patients with newly diagnosed acute myeloid leukemia: 20.0%	[266]
Gemtuzumab ozogamicin	Complete remission in patients with a median age of 61 years old: 30%	[267]
Gilteritinib	Complete remission with full or partial hematologic recovery in patients with FLT3 mutations: 34.0%	[253]
Low-dose cytarabine	Complete remission for patients >70 years old: 7.0%	[268]
Midostaurin	Complete remission in patients between 18 to 59 years old, and FLT3 mutations: 58.9%	[18]
Venetoclax	Complete remission + complete remission with incomplete hematological recovery: 54% (in combination with low-dose cytarabine)	[269]
Venetoclax + low-dose cytarabine	Complete remission/ Complete remission with incomplete blood count recovery for patients >60 years old: 54.0%	[270]

## Data Availability

Not applicable.
